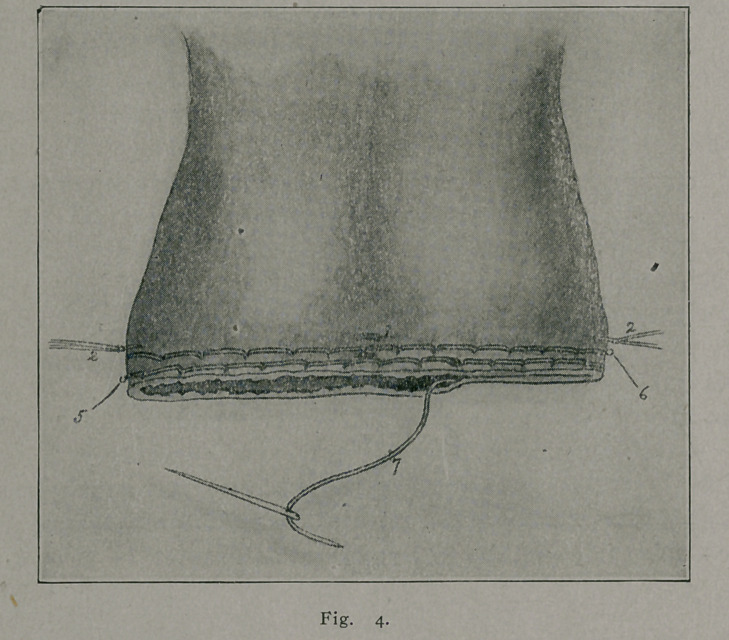# Varicocele in the Male. (Part 2

**Published:** 1911-03

**Authors:** Aime Paul Heineck

**Affiliations:** Surgeon to the Grace, Reliance and Cook County Hospitals; Senior Prof. of Surgery, Reliance Med. Coll.; Adj. Prof. of Clinic Surgery, Coll. of Physicians and Surgeons, University of Ills., Chicago, Ills.


### VARICOCELE IN THE MALE.

### *(a) Discussion of the Condition. (b) A New Operative Procedure for Its Successful Treatment.*

By Aime Paul Heineck, M. D.

*Surgeon to the Grace, Reliance and Cook County Hospitals; Senior Prof. of Surgery, Reliance Med. Coll.; Adj. Prof. of Clinic Surgery, Coll. of Physicians and Surgeons, University of Ills., Chicago, Ills.*

*(Continued from February Issue.)*

For the treatment of varicocele, many various operative procedures have been suggested. Vince (31), treats varicocele by resecting and shortening the lengthened and relaxed cremaster muscle. He incises the skin from the external abdominal ring to the superior pole of the testicle. An incision of the same length divides the intercolumnar fascia and the cremasteric fascia and muscle longitudinally. The cord is elevated from its bed and retracted. Vince then applies transversely on the cremaster muscle two forceps at a distance of 6 cm. from each other and resects that portion of the muscle extending between the forceps. The two muscular extremities having been sutured to each other, the cord is replaced on the surface of the muscle, and the longitudinal incision closed. In exceptional cases, Vince supplements this procedure by partial resecting of the diseased veins.

Brault (24), resects the varicose veins and, in addition, excises an oval flap from the postero-external surface of the scrotum. He recommends the employment of his method in all cases of varicocele that have recurred after bilateral resection of the scrotum. For the operative treatment of varicocele he considers bilateral resection of the scrotum the operation of choice.

Brault's operation consists of the following steps:

1. Excision of an oval postero-external flap.
2. Longitudinal division of the spermatic cord's sheaths.

We aim by these two operative procedures, performed at one and the same sitting:

1. To suppress the subjective symptoms: pain, sensation along the inguinal canal, etc.

2. To secure the re-establishment to physiological conditions of the altered venous circulation and thereby to prevent degenerative changes in the testis. Should the testis be undersized or somewhat atrophied, call, previous to operation, the patient's notice to the condition; it becomes more apparent after resection of the veins.

3. To restore to the scrotum its normal contour and dimensions.

4. To support the testicle in such a way as to permanently hinder its descent as well as to prevent the elongation of the spermatic cord.

5. The removal in part of the diseased vessels.

In the operative treatment which we practice and recommend for varicocele, we make a direct and an indirect attack upon the existing pathological conditions. We ablate some of the varicosed veins, we shorten the relaxed and lengthened scrotum. It is a mixed method suppressing by resection of the varicosed veins the main element of the condition, and by resection of the pendulous scrotum, an accessory, a contributory element of great importance.

This double operative procedure:

(a.) Resection in part of the diseased veins,

(b) Resection of the pendulous and attenuated scrotum,—can, without haste, be readily performed in about 15 minutes. It entails no risks to life and, when performed by careful and experienced hands, is never followed by undesirable, immediate or remote sequelae. An assistant is necessary.

The patient and the operative field having been prepared according to the teachings of modern, aseptic surgery as for a major operation, it is well to have recourse to general anaesthesia. We know that these operations can be and have been performed successfully with the aid of local anaethesia, but clinical observation and operative experience have taught us they can be performed immeasurably better if the patient be anaesthetized by the aid of a general anaesthetic. General anaesthesia secures a more complete abolition of pain and enables the surgeon to do his work deliberately and precisely.

3. Resection of the varicosed spermatic vessels.

4. Careful suturing of the sheaths of the spermatic cord.

5. Closure of wound in such a way that the resulting line of suture has the shape of an inverted V.

For the cure of varicocele, Parona (18), has devised an operation that still enjoys a degree of popularity. Its different steps of execution are the following:

1. Make a 6 cm. incision, extending from the external abdominal ring downward upon the neck of the scrotum.

2. Isolate the testicle. The testicle and the spermatic cord are completely freed so as to permit their delivery, their enucleation through the scrotal incision. The cord is isolated as far as the external abdominal ring.

3. Incise longitudinally and then evert the tunica vaginalis testis. After eversion, the upper margin of the tunica vaginalis is sutured with cat-gut to the internal pillar, to the pubic fibrous tissue and to the external pillar in such a way as to convert the vaginal tunic into a sac ensheathing the dilated veins. The empty scrotal sac created by the suspension of the testicle is obliterated by suturing the opposed walls.

Parona aims by this approximation of the testicle to the external abdominal ring:

1. To lessen the height and weight of the blood column in the spermatic vein and branches.

2. To favor the venous return circulation.

3. To aid the action of the cremaster muscles.

4. To obtain a permanent firm physiological suspension of the testicle. The everted and fixed tunica vaginalis maintains the testicle elevated, exerts moderate compression upon the varicose veins and to a degree hinders the elongation of the spermatic cord. Parona's operation is not serviceable in the presence of a markedly pendulous scrotum or of a varicocele too voluminous to be contained in the vaginal suspensory. It has been objected to Parona's operation that insomuch as it deprives the testicle of its vaginal envelope it is anti-physiological.

Fig. 1.

Fig. 2.

Fig. 3.

Fig. 4.

The reference numbers apply to any of the figures.

1. Ligatures perforating anterior and posterior scrotal walls in the region of the median raphe.

2. Right and left lateral ligatures knotted and temporarily left long, to be held taut and serve as guy-ropes during introduction of the two suture ligatures.

3. Suture ligature showing method of introduction and how it includes within its loops the tissues comprising the scrotal walls.

4. Line of scrotal section.

5. First suture-ligature inserted at about 1-4 cm. from proposed line of scrotal section.

6. Second suture-ligature inserted at about 1-4 cm. from proposed line of scrotal section. Note that needle punctures of upper suture-ligature correspond to about the middle of the loops of lower suture-ligature

7. Subcuticular stitch approximating edges of scrotal wound.

8. Forceps helping to spread out scrotal tissues.

Though the fore-mentioned methods have, in some hands, given good results, we recommend their general abandonment and the employment of the operative procedures, separately exceptionally, conjointly almost always, that we are about to describe.

### Operation Proper.

1. Patient in the dorsal recumbent posture, the lower limbs straight out, short distance apart.

2. Repreparation of the operative field: inguinal, pubic and scrotal regions.

3. The operator makes an inch or an inch and a half oblique incision, the mid-point of which corresponds to the pubic spine, dividing the skin and superficial fascia and exposing the spermatic cord. This incision is practically a suprapubic incision. It is easier to isolate the veins close to the inguinal canal than near the testis, as here fewer vessels have to be ligated, the mass included in the ligature is smaller. Thompson says that the secret of the operation is to attack the veins high up where they are lying in a distinct tube of fat and fascia, distinct from the vas.

4. The spermatic cord is then isolated and elevated from its bed. The cord's envelopes, the infundibuliform fascia, the cremasteric fascia and muscle, and the intercolumnar fascia are incised longitudinally and thus the spermatic veins and branches are made easily accessible.

5. Identify the vas deferens and if possible the spermatic artery (38.) The vas deferens, owing to its volume, its consistency, and its cord-like feel, can always be recognized; the spermatic artery, however, is at times extremely difficult to positively identify. As the pulsations of the spermatic artery are often imperceptible, they do not furnish a constant guide to the vessel. Bear in mind that the artery is always close to the vas deferens, that it accompanies it and follows the same course, and avoid including the vessel in the ligatures. (38.) Do not injure the vas deferens and its blood supply. Leave the veins of the vas deferens and also those that course upon the cord's sheaths undisturbed. These vessels should not be ligated, should not be resected, as they are important for the re-establishment of the collateral circulation. The spermatic veins have numerous anastomoses with the veins of the vas deferens, of the scrotum, of the septum scroti. Operate with as little traumatism as possible, and observe the most rigorous asepsis. Let there be no needless handling of the vas deferens, of the epididymis, of the testis. If the vas deferens or testicle be roughly handled, orchitis or epididymitis may supervene.

6. The condition is usually limited to the spermatic veins or pampiniform plexus. The larger portion of this plexus can be resected. To resect all of the veins of the spermatic cord is a grave mistake. In Porter's (32), case, after an operation for varicocele, the testicle, owing to a sufficient blood-supply not having been left, became inflamed, was unable to recover, and sloughed.

Isolate the veins for a greater distance than the amount of vessels to be removed, so that when divided ends are united too great kinking of the vas will not take place. (33.) Though the vas deferens is about 18 inches long, the actual distance traversed by it is, owing to its somewhat convoluted course, not more than 12 inches. Therefore, shortening of the cord by resection of the veins does not interfere with the functions of the vas deferens. Most operators ligate the veins with strong catgut at two different points, about two inches apart. The intervening portion of the vein is resected. Other operators ligate the veins about half an inch above the epididymis, and again a little below the external abdominal ring and resect the intervening portion. It goes without saying that these compressing ligatures are applied perpendicularly to the course of the vessels. The upper and lower ligatures are tied to each other, there results from this apposition of the ends of the severed veins an induration which need cause no alarm, as it gradually undergoes absorption in about three months. (Potter, 35.)

The ligation and resection of the left spermatic veins interrupts the weight of the venous blood column that formerly extended from the renal vein downwards to the testicle. The knotting together of the upper and lower ligatures of the divided veins assists the enfeebled cremaster muscle in its endeavors to support the dependent testicle. This also removes more or less continuous strain from the vas deferens, and its accompanying vessels. After approximating the ligatures, the proximal and distal stumps are sutured to each other.

Eads (34), and others advise avoiding injury to the genito-crural nerve which supplies the cremaster muscle. If this nerve is cut, the portion of the cremaster muscle distal to the seat of the division is deprived of its power of contractility, its blood-supply is diminished, it wastes, weakens, stretches and the natural consequence is a flabby scrotum.

7. Carefully inspect the stumps for oozing. Great care must be taken to secure complete hemostasis, for small bleeding points may give rise to large-sized hematomata. Slight hemorrhage, such as would occur in connection from a damaged vein leads to the formation of a hematoma which can, by exerting pressure upon the remaining veins, prove a potent factor in determining oedema and thickening of the scrotum, subjacent tissues and testis. Post-operative hemorrhage may be due to slipping of the ligature, to the use of a faulty knot, to defective ligature material.

By tying together the proximal and distal ends of the divided vessels, in case of slipping of ligatures, it is easier to locate the bleeding point. Krone (36), anchors the divided stump of veins above, to fibers of ring, and below, to Poupart's ligament.

Corner and Nitch (37), report two cases of varicocele in which resection of the veins was followed by post-operative hemorrhage. In these two cases the pelvis was filled with blood which had escaped from the retracted end of the spermatic artery projecting through a rent in the peritoneum.

8. After all hemorrhage has been arrested, the divided sheaths of the cord are sutured and this is followed by the closure of the operative wound.

As previously stated, we always supplement this resection of the veins of the spermatic cord by partial amputation of the scrotum. We consider this step essential to effect a prolonged, if not a permanent, cure of the condition. In over one hundred cases operated on during the last two years at the West Side, Reliance, University, and Cook County Hospitals, we have not noted a single tendency to recurrence.

The relaxed pendulous and attenuated state of the scrotum associated with varicocele suggests retrenchment of the redundancy. By resection of the scrotum, a natural suspensory is formed which will keep the testicles in good position and prevent a recurrence of the disease. A close fitting scrotum, by better supporting the testes, by keeping them higher, prevents traction of the veins of the pampiniform plexus and thus renders them less diable to dilatation.

Skin of the scrotum is thin, elastic, is pigmented and marked by longitudinal raphe and when contracted by transverse ridges. In scrotectomy performed secundum artem, the vas deferens and its vessels and the spermatic artery are not exposed to injury.

The technique for scrotectomy which we are about to describe, possesses the following advantages:

1. Rapidity and simplicity of execution. Interrupted sutures are not used, they complicate and prolong the operation and do not afford as much protection against hemorrhage as the continuous suture-ligatures employed.

2. Adaptability to the cure of relaxed scrotum irrespective of cause. It will be found serviceable to correct scrotal overdistention caused by voluminous varicoceles, large scrotal hernias, large hydroceles, testicular neoplasms, etc. It builds out of the scrotal envelopes a natural suspensory and removes all the scrotal tissue that appears needless, superfluous.

3. It requires little if any post-operative treatment. As cat-gut is the only suture—and ligature—material used, there is no call for the removal of stitches or ligatures. The portion buried in the tissues is absorbed, the remaining portion is cut off.

4. No special instrument is required. No clamps are used. Two needles, three artery forceps and a pair of scissors suffice to accurately perform the operation.

5. Absolute control of operative hemorrhage.

6. Absolute control of post-operative hemorrhage.

7. Safety and efficacy. In over one hundred cases, our results have been uniformly good. We have had a few cases of healing by delayed first intention, but, in these cases, even healing by secondary intention does not unfavorably influence the ultimate results of the operation.

Scrotectomy would have enjoyed a greater popularity if a method had been devised previous to our own enabling the surgeon in this operation to easily and surely control hemorrhage. It is the fear of hemorrhage, operative and post-operative, the fear of hematoma formation which has deterred many surgeons from performing this operation, and which has led others to accident. There is not any clamp, whether convex or concave, whether designed to be applied proximally or distally to the site of section, that has proved universally efficient. It is now conceded that clamps do not furnish an absolute safeguard against hemorrhage. Accidents have followed their use by competent hands. (Dardignac, Lucas Championniere, etc.) We have discarded the use of clamps, special or others, and have succeeded in working out a technique which absolutely eliminates all danger of hemorrhage, primary or secondary.

In resecting a scrotum, the line of section may be unilateral, may be bilateral, may be longitudinal, may be transverse. We almost always resort to a bilateral transverse line of section. The same technique, however, is serviceable for a longitudinal line of section. In longitudinal resection, the cicatrix falls in the line of the median raphe, or rather reconstitutes it, and the scrotum is in no way deformed. Transverse bilateral resection possesses the advantage of acting upon both halves of the scrotum at the same time, and of giving a cicatrix that does not in any way interfere with future penile erections.

Proceed as follows:

1. The assistant with the fingers of one hand spreads the scrotum to its maximum, and with the fingers of the other hand pushes the testes towards the inguinal canal. It is desirable that neither the testes nor the tunicae vaginalis be traumatized or injured. The operator then estimates the amount of scrotal tissue which it is proper to remove. Enough must be removed so that the new scrotal sac will firmly support the testes. Care must also be taken not to remove too much, otherwise, the new scrotal sac will cause discomfort by compressing the testicles against the pubic bones.

2. It has been observed in this operation that the vessels of the septum scroti were frequently the origin of the post-operative hemorrhage. Therefore, in scrotectomy, these vessels must be kept in mind. In operative surgery, the customary and elective way of arresting hemorrhage is by ligating vessels at their bleeding points. Surgeons rarely depart from this rule, and the ligation in continuity of a vessel for the arrest of hemorrhage is an exceptional procedure performed only under exceptional conditions. In the ligation of a vessel, the compressing ligature is placed perpendicularly to the course of the vessel and directly upon its walls. This is the usual procedure and is known as immediate ligation. In scrotectomy, however, we make use of immediate ligation, the compressing loop of cat-gut is placed perpendicularly to the long axis of the vessel, and in such a way that between it and the vessel wall there intervenes a layer of scrotal skin and underlying tissues.

3. Cat-gut ligatures are introduced at the point marked 1 (Figs. 1, 3 and 4), they are knotted and cut short. These ligatures perforate the anterior and posterior scrotal walls at about the median raphe and are designed to control, to prevent hemorrhage from the septal regions. They are important factors in the securing of hemostasis.

4. One ligature is introduced at each lateral margin of the scrotum (2 Figs. 1, 3 and 4). These two ligatures are knotted, and the ends for the time being left long serve as guy-ropes, maintaining the scrotal tissues taut while the two suture-ligatures are being introduced.

5. The point of scrotal resection has previously been determined (4 Fig. 1, 3 and 4.) Two long ligatures of thick catgut are selected and each one is needled at both ends. The needles which I prefer for these suture-ligatures are long straight needles, flattened from side to side (straight spear-pointed needles are also useful). No needle-holder is used. The needle-eyes must be large enough to allow the easy gliding into them of the catgut. The assistant, by the aid of the two lateral ligatures (2 Figs. 1, 3 and 4), and a forceps or tenacula placed at point 8 (Figs. 1 and 3), spreads out fan-shaped the portion of scrotal tissue which the surgeon is about to ablate.

6. At about a 1-4 cm. from the proposed line of scrotal section, the operator makes the middle of one of the double-needled strands of cat-gut saddle the lateral scrotal margin nearest to him, and then proceeds with the introduction of the first suture-ligature as shown in plate 3. This is a continuous stitch somewhat analogous to the cobbler's stitch, extending from one lateral scrotal margin to the other and including in its loops the anterior and posterior scrotal walls and intervening tissues (5 and 6, Figs. 1, 3 and 4.) It is seen that the two needles are used at the same time; and that they constantly go in diametrically opposite directions (Fig. 2). Upon reaching the further lateral margin, the ends of the suture ligature are tied, knotted and cut short.

7. A similar continuous, cobbler-stitch-like suture-ligature, extending from one lateral scrotal margin to the other, is now inserted (6 Fig. 3 and 4), at about a 1-4 cm., within the one just introduced, or at about 1-2 cm. within the line of proposed scrotal section (4 Figs. 1, 3 and 4). Like its mate, it perforates the anterior and posterior scrotal walls and its loops are extended to approximate the scrotal tissues and to control hemorrhage. By looking at the illustrations it will be seen that the needle punctures of one suture-ligature correspond to the middle of the loops. After this suture-ligature has covered the entire transverse width of the scrotal sac, it is tied, knotted and its ends are cut short.

8. The operator now cuts off with scissors the redundant scrotal tissue. 4 corresponds to the line of scrotal section.

9. Usually the edges of the wound gape and this is overcome by the introduction of a continuous subcuticular cat-gut stitch. (7 Fig. 4). The wound is dressed, rubber tissue being placed over the dressing to prevent the possibility of contamination by urine.

10. A double spica gauze-bandage is now so applied as to maintain the testicles elevated upon the abdomen, and to exert slight but painless compression upon the new-formed scrotal sac. As after other operations performed upon the spermatic cord or the scrotum, the patient may suffer for a few days from urinary retention. This is easily and safely overcome by gentle and aseptic catheterization.

Resection of veins is occasionally followed by some oedema of the scrotum, a little engorgement of the testes, and a moderate effusion into tunica vaginalis. This gradually disappears and need cause no alarm.

So as to maintain the operative region dry, it is well to change the scrotal bandage every few days.

The patient is confined to bed two weeks and for a month thereafter, but no longer, is to wear a well-fitting suspensory.

### REFERENCES.

1. Quain, Richard.—A Dictionary of Medicine, 1894, vol. 2, p. 1158. D. Appleton & Co., N. Y.

2. Senn, N.—On the Frequency of Varicocele and the Limitations of Operative Treatment for this Affection. Phil. Med. Journ., 1898, vol. 1, p. 1165.

3. A. Pearce Gould.—Two Cases of Varicocele with Undeveloped Testicle, with Remarks on the Nature of Varicocele. Clin. Society's Transactions, 1881, vol. 14, p. 75.

4. Chassaignac, Chas.—Med. Rec., 1902, vol. 62, p. 603.

5. Dardignac, J. J. A.—Note sur le varicocele et son traitement, Revue de Chirurgie, 1895, vol. 15, p. 721.

5a. Istomin, E. K.—Zur pathologischen Histologie und Klinik der Varikokele, Deutsche Zeitschrift feur Chirurgie, 1909, p. 1, vol. 99.

6a. Rocher.—Varicocele infantile, J. de Med. De Bordeaux, 1906, vol. 36, p. 648.

6b. Broca, A.—Varicocele chez l'enfant, Le Bull. Mel., 1902, vol. 16, p. 985.

7. Curling, T. B.—On Diseases of the Testis, 1878. Lindsay & Blakiston, Phil.

8. Hochenegg, J.—Ueber Diagnose und klinische Beduetung der symptomatischen Varikokele bie malignen Nierentumoren, Zeitschr. fuer Klin. Medezin, 1907, vol. 62, p. 51.

9. Duplay & Reclus.—Traite de Chirurgie, Paris, 1899, vol. 7, p. 1215.

10. Delbet, P.—Cancer du fois secondaire a un epithelioma juxta-pylorique de l'estomac demeure latent. Coexistence d'un varicocele symptomatique a droite, Bull. & Mem. de la Societie Anatomique de Paris, 1901, vol. 3, p. 461.

11. Longuet L. Chirurgie reparatrice du varicocele, La Presse Med., 1902, vol. 10, p. 879.

12. Deschamps, H.—Les notions nouvelles sur le varicocele. Prog. Med., Paris, 1906, 3 ieme serie, vol. 22, p. 131.

13a. Corner, E. M.—Varicocele—What of It? Lancet, 1905, vol. 2, p. 993.

13b. Howard, Frank.—Varicocele—What of It? Lancet, 1905, vol. 2, p. 923.

14. Boland, Frank K.—Varicocele and Its Operation. Journ A. M. A., 1908, vol. 50, p. 1888.

15a. Dudley, A. P.—Varicocele in the Female, What Is Its Influence upon the Ovary? N. Y. Med. Journ., 1888, vol. 48, p. 147, 174, 183.

15b. Miller & Kanavel, Uncomplicated Varicose Veins of the Female Pelvis, Amer. Journ of Obst., 1905, vol. 51, p. 480.

15c. Shober, John B.—Varicocele of the Broad Ligament, Amer Journ. of Obst., 1901, vol. 43, p. 664.

16. Picque, Lucien, Varicocele et Obsession, Ann. des Maladies des Organes Genito-Urinaires, Paris, 1905, vol. 23 p. 1169.

17a. Carta, F. Semaine Med., 1908, vol. 28, p. 606.

17b. Narath, Radikal Operation der Varikokele, Wiener Klin. Wochenschr. 1900, vol. 13, p. 73.

18. Spillman, G., Note sur quatorze observations personnelles de cure chirurgicale du varicocele par le procede de Parona, Arch. Prov. de Chir., 1902, vol. 2, p. 221.

19. Patel & Charlier, Les tumeurs du cordon spermatique, Revue de Chir., 1909, vol. 39, p. 119.

20a. Burghard, F. F.—A System of Operative Surgery. Oxford University Press, 1909, vol. 3, p. 639.

20b. Longuet, L.—Un cas de thrombophlebite du cordon traite par la phlebectomie, La Presse Med., 1899, vol. 7, p. 166.

21. Patel, M.—Rupture de varicocele, Ann. des Maladies des Organes Genito-Urinaires, Paris, 1904, vol. 22, p. 1521.

22. Death from Rupture of a Varicocele, The Lancet, 1860, vol. 1, p. 295.

23. Lomeau, Guerison operatoire d'un enorme varicocele, Gaz. Hebd. des Sciences Med. de Bordeaux, 1903, vol. 24, p. 186.

23a. Loison.—Traitement du varicocele par le procede de Narath, Bull. & Mem. de la Societe de Chirurgie, 1900, vol. 26, p. 636.

24. Brault, J.—Excision postero-laterale du scrotum combinee avec la resection des veins dans les varicoceles compliques, Bull. & Mem. de la Soc. de Chir., 1900, vol. 26, p. 704.

25. Aquirre, A.—Journ. of the Assoc. of Military Surgeons of the U. S., 1905, vol. 16, p. 46.

26. LeFort.—Gaz. des Hospitaux de Toulouse, 1901, 15 ieme annee, p. 222.

27. Heineck, A. P.—Modern Surgical Treatment of Exophthalmic Goitre Ills. Med. Journ., February, 1908.

28. Heineck, A. P.—The Modern Operative Treatment of Fractures of the Patella, Surg., Gynec. & Obst., 1909, vol. 9, p. 177.

29. Lydston, G. Frank.—Radical Treatment of Varicocele, Alkaloid Clinic, Chicago, 1901, vol. 8, p. 449.

30. Lewis, Dean D.—The High Operation for Varicocele, Surg., Gynec. & Obst., Chicago, 1906, vol. 3, p. 534.

31. Vince.—Nouveau procede de cure chirurgicale du varicocele, Journ. Med. de Brux, 1904, vol. 9, p. 525.

32. Porter, F. J. W.—Operation for Varicocele, Sloughing of the Testicle during Convalescence from Enteric Fever. Brit. Med. Journ. 1903. vol. 2, p. 134.

33. Furniss, H. D.—Varicocele, Amer. Med., Phil., 1904. vol. 7, p. 891.

34. Eads, P. Brindley.—Demonstration. Med. Standard, Chicago, 1904, vol. 27, p. 521.

35. Potter, E. S.—Suprascrotal Operation for Varicocele, with Ligature of the Spermatic Artery. N. Y. Med. Journ., 1903, vol. 77, p. 789.

36. Krone, C. R.—Suprabubic Varicocele-Ectomy, Occidental Medical Times, Sacramento, 1898, vol. 12, p. 301.

37. Corner & Nitch.—The Immediate and Remote Results of the High Operation for Varicocele. Brit. Med. Journ., London, 1906, vol. 1, p. 191.

38. Griffiths, Joseph.—The Effects upon the Testes of Ligature of the Spermatic Artery, Spermatic Veins, and of Both Artery and Veins. Journ of Anatomy and Physiology, 1895-1896, vol. 30, p. 80.

## Figures and Tables

**Fig. 1. f1:**
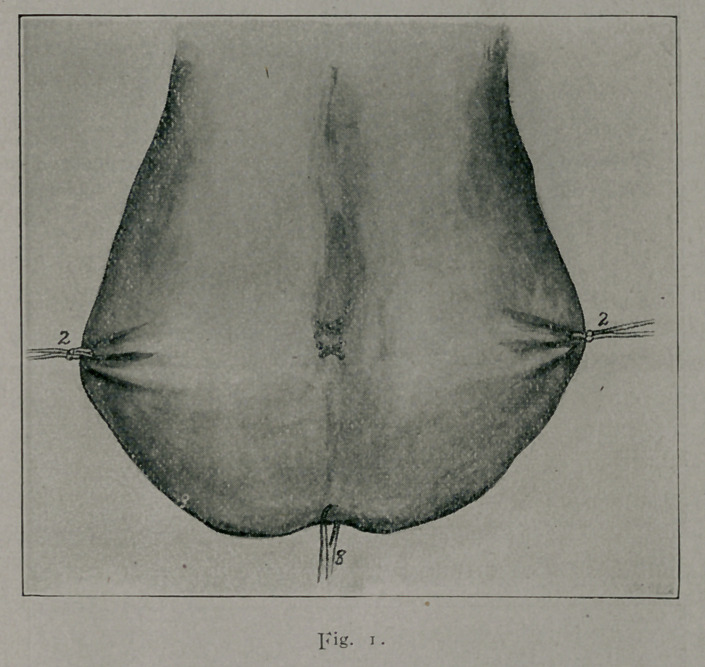


**Fig. 2. f2:**
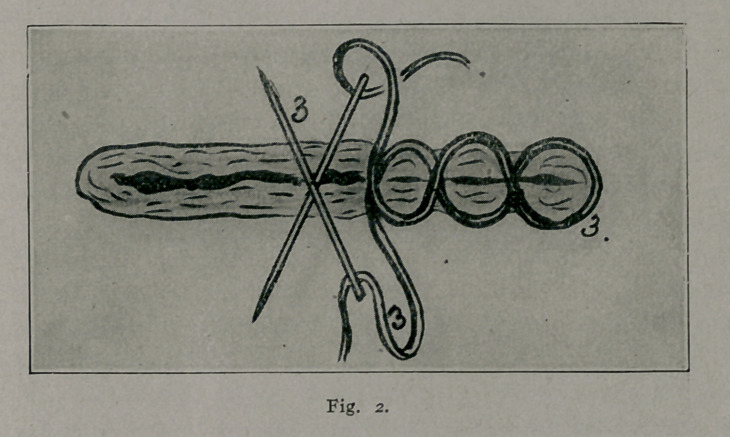


**Fig. 3. f3:**
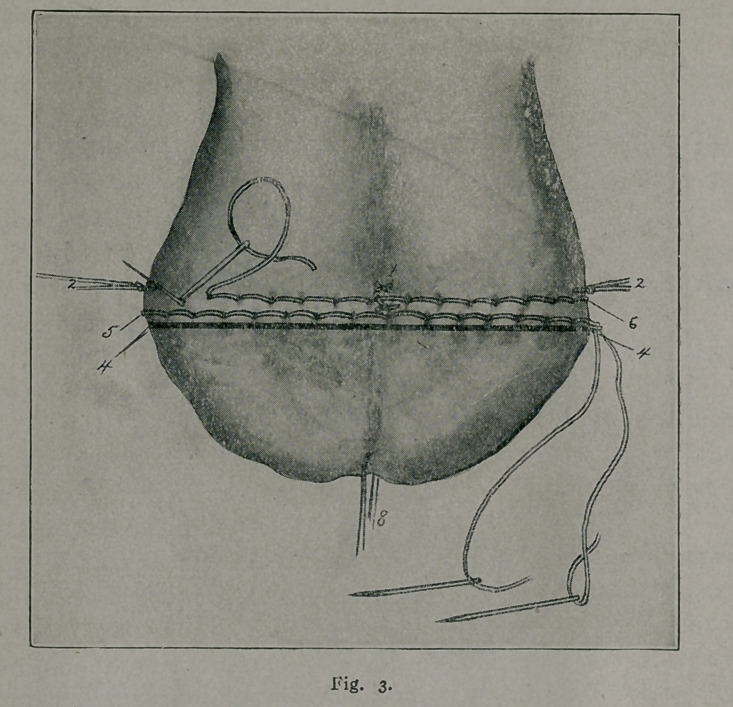


**Fig. 4. f4:**